# Reported Knowledge of Recent HbA1c, Blood Pressure, and LDL-cholesterol Levels and Goals Among US Adults with Diabetes—National Health and Nutrition Examination Survey 2011–2020

**DOI:** 10.1007/s11606-026-10474-8

**Published:** 2026-05-06

**Authors:** Bharat Poudel, Ligong Chen, Medha J. Dubal, Ashley E. Christenson, Caroline Presley, Paul Muntner, Michael Fang, Kathryn Foti

**Affiliations:** 1 Department of Epidemiology, School of Public Health, University of Alabama at Birmingham, Birmingham, AL USA; 2 Perisphere Real World Evidence LLC, Austin, TX USA; 3 Johns Hopkins Bloomberg School of Public Health, Baltimore, MD USA; 4 Department of Medicine, Heersink School of Medicine, University of Alabama at Birmingham, Birmingham, AL USA

**Keywords:** knowledge, diabetes, HbA1c, blood pressure, LDL-cholesterol

## Abstract

**Background:**

Guidelines recommend adults with diabetes should know their hemoglobin A1c (HbA1c), blood pressure (BP), and low-density lipoprotein (LDL)-cholesterol levels and goals.

**Objective:**

Determine the proportions of US adults with diabetes who report knowing their HbA1c, BP, and LDL-cholesterol levels and goals as recommended by their doctor or healthcare provider.

**Design:**

A cross-sectional analysis of the US adults with self-reported diabetes from 2011–2020 in the National Health and Nutrition Examination Surveys (NHANES).

**Participants:**

US adults in NHANES with self-reported diabetes.

**Main Measures:**

Sociodemographic characteristics including age, sex, race and ethnicity, education, and health insurance coverage were assessed using standardized questionnaires. The outcomes were self-report of knowing HbA1c, BP, and LDL-cholesterol levels and goals. Age-sex-adjusted Poisson regression models with robust variance estimation were used to estimate associations of participant characteristics with each of these outcomes.

**Results:**

Among US adults with diabetes included in the analysis (*N* = 3,464), 58.3% (95% CI: 55.4%-61.2%), 68.3% (95% CI: 65.7%-70.7%) and 18.0% (95% CI: 16.3%-19.8%) reported knowing their recent HbA1c, BP, and LDL-cholesterol levels, respectively, and 53.4% (95% CI: 50.5%-56.2%), 39.7% (95% CI: 37.2%-42.3%), and 13.4% (95% CI: 11.5%-15.6%) reported knowing their HbA1c, BP, and LDL-cholesterol goals, respectively. Non-Hispanic Black and Hispanic versus non-Hispanic White adults, those with less than versus at least a high school education, and those without versus with health insurance were less likely to report knowing their HbA1c, BP, or LDL-cholesterol levels and goals.

**Conclusion:**

Low percentages of US adults with diabetes reported knowing their HbA1c, BP, or LDL-cholesterol levels and goals. Understanding reasons for the gaps in knowledge among US adults with diabetes may facilitate interventions to increase risk factor awareness and diabetes self-management behaviors.

**Supplementary Information:**

The online version contains supplementary material available at https://doi.org/10.1007/s11606-026-10474-8.

Among adults with diabetes, higher levels of hemoglobin A1c (HbA1c), blood pressure (BP), and low-density lipoprotein (LDL) cholesterol are each associated with an increased risk of microvascular and cardiovascular disease (CVD).^1,2^ Studies have shown that lowering HbA1c, BP, and LDL-cholesterol reduces diabetes complications and CVD.^3^ However, in 2015–2018, only 50.5% of US adults with diabetes achieved glycemic control as recommended by the American Diabetes Association (ADA) [HbA1c < 7%, 53 mmol/mol], while 70.4% had controlled BP (systolic BP < 140 mm Hg and diastolic BP < 90 mm Hg), and 60.3% had LDL-cholesterol < 100 mg/dL.^4^

Diabetes self-management education and support (DSMES) is a cornerstone of diabetes care.^5^ For adults with diabetes, knowing their HbA1c, BP and LDL-cholesterol numbers and levels recommended by their health care provider may reflect patient-provider communication, as well as patient engagement, and may facilitate self-management behaviors.^5^ Professional societies including the ADA and American Heart Association (AHA) recommend individuals with diabetes know their HbA1c, BP, and cholesterol levels.^5,6^ A prior study among patients with diabetes from five health systems found that 66% of those who had their HbA1c checked in the past six months did not know their last HbA1c value.^7^ Other studies have shown gaps in reported knowledge of HbA1c, BP, and LDL-cholesterol levels and goals among US adults with diabetes, and that reported knowledge is higher for HbA1c than BP or LDL-cholesterol.^8,9^ Determining the percentages of adults with diabetes who report knowing their recent HbA1c, BP, and LDL-cholesterol levels and goals based on updated population-based data and identifying associated characteristics may help inform directed interventions.

The goal of the current study was to estimate the percentages of US adults with diabetes who reported knowing their recent HbA1c, BP, and LDL-cholesterol levels and goals using data from the National Health and Nutrition Examination Survey (NHANES). Also, we examined factors associated with reported knowledge of HbA1c, systolic and diastolic BP, and LDL-cholesterol levels and goals.

## RESEARCH DESIGN AND METHODS

### Data Source

The NHANES is a program conducted by the National Center for Health Statistics (NCHS) of the US Centers for Disease Control and Prevention (CDC), which was designed to monitor the health of the US population through a series of cross-sectional surveys. Using a complex, stratified, multistage probability-cluster sampling design, NHANES participants were selected from the US non-institutionalized, civilian population. We pooled the data from four NHANES cycles, 2011–2012, 2013–2014, 2015–2016, and 2017-March 2020. We used data through 2020 as the 2021–2023 NHANES cycle did not collect information on reported knowledge of recent HbA1c, BP, or LDL-cholesterol levels and goals. The NHANES protocols were approved by the NCHS institutional review board and written informed consent was provided by all participants.

### Study Population

There were 42,295 NHANES 2011–2020 participants who completed an interview and examination, of whom 26,239 were 18 years of age or older (Supplemental Fig. 1). We restricted the study population to participants who were not pregnant (*N* = 25,960). We further restricted the study population to adults who were aware that they had diabetes, defined by a self-report of a prior diagnosis of diabetes by a doctor or healthcare provider (*N* = 3,518). We additionally excluded participants who had missing information on questions asked about their recent HbA1c, BP and LDL-cholesterol levels and goals. The final analytic sample included 3,464 NHANES participants.

### Data Collection

Participant data were collected during an in-home interview and an examination visit which took place at a mobile examination center. The examination included physical measurements, including BP and blood and urine collection. Of relevance to the current analysis, blood and urine were used to measure serum creatinine, HbA1c, total and HDL cholesterol, serum triglycerides, and urinary albumin and creatine. Sociodemographic characteristics including age, sex, race and ethnicity, education, and health insurance coverage were assessed using standardized questionnaires. Participants self-reported how long they had diabetes and history of CVD, which was defined by a prior diagnosis of myocardial infraction, coronary heart disease, heart failure, or stroke. Chronic kidney disease (CKD) was defined as an estimated glomerular filtration rate < 60 mL/min/1.73m^2^ using the 2021 CKD-Epidemiology Collaboration Eq. ^10^ or a urinary albumin-to-creatinine ratio > 30 mg/g.

### Blood Pressure, HbA1c, and LDL-Cholesterol Measurements

BP was measured using a mercury sphygmomanometer with standard Bauman cuffs in NHANES cycles conducted in 2011–2012 through 2015–2016. In NHANES 2017–2020, BP was measured using a validated oscillometric device. As in prior analyses of NHANES data, we calibrated the oscillometric device values to the mercury device values by adding 1.5 mm Hg to oscillometric-measured systolic BP and subtracted 1.3 mm Hg from oscillometric-measured diastolic BP for participants in NHANES 2017–2020.^11^ In all NHANES cycles, BP was measured following a standardized protocol by a physician who completed training and certification. Systolic and diastolic BP were measured three times after participants were seated and rested for a minimum of five minutes. The mean of the measurements was used to define systolic and diastolic BP. Hypertension was defined as systolic BP ≥ 140 mm Hg, diastolic ≥ 90 mm Hg, or self-reported use of antihypertensive medication.^12^ Both HbA1c and LDL-cholesterol levels were assessed using blood collected during the study examination. LDL-cholesterol was calculated among a sample of participants who were asked to fast overnight prior to their study visit (N = 950) using the Sampson equation: LDL-cholesterol = Total cholesterol/0.948 – high density lipoprotein cholesterol/0.971-(triglycerides/8.56 + triglycerides * non-high density lipoprotein cholesterol/2140- triglycerides * triglycerides/16100)−9.44.^13^ We defined glycemic control as HbA1c < 7%, BP control as systolic BP < 140 mm Hg and diastolic BP < 90 mm Hg, and LDL-cholesterol control as LDL < 100 mg/dL.

### Outcome Variables

A detailed definition of outcome variables is presented in Supplemental Table 1. Reported knowledge of recent HbA1c, BP, or LDL-cholesterol levels and goals was defined by providing a numeric value within clinically plausible range as provided in the NHANES questionnaire. NHANES did not assess the accuracy of participant-reported levels and goals. Therefore, the outcome assesses whether participants reported knowing their risk factor level and goals rather than their accuracy. The participant’s response was recorded as not knowing the level or goal if the response was “Refused,” “Don’t know,” or “Provider did not specify goal.” We defined reported knowledge of BP levels and goals based on having a response for both systolic and diastolic BP.

### Statistical Analysis

Descriptive statistics were calculated using percentages (95% confidence interval [CI]) for categorical variables and mean (95% CI) for continuous variables. We calculated the percentage (95% CI) of US adults with diabetes who reported knowing their recent HbA1c, BP, and LDL-cholesterol levels and goals. We examined the distributions of self-reported HbA1c, BP, and LDL-cholesterol levels and goals. Poisson regression with robust variance estimation was used to estimate the age and sex adjusted prevalence ratio (PR) and 95% CI for knowing versus not knowing recent HbA1c, BP, LDL-cholesterol levels, separately, for each participant characteristic.

Additionally, we calculated the mean HbA1c, systolic and diastolic BP, and LDL-cholesterol among those who reported knowing and not knowing their respective risk factor levels and goals. We also calculated the percentage with control of HbA1c, BP, and LDL-cholesterol among those who reported knowing and not knowing their respective risk factor levels and goals. We used linear regression to calculate the age and sex adjusted difference in mean HbA1c, systolic and diastolic BP, and LDL-cholesterol comparing those who did and did not report knowing their recent HbA1c, systolic and diastolic BP, and LDL-cholesterol levels and goals, separately. We used Poisson regression with robust variance estimation to estimate the age and sex adjusted PRs (95% CI) for the association between reported knowledge of HbA1c, BP, and LDL-cholesterol levels and goals with control of these risk factors, separately. Additionally, we conducted linear and Poisson regression models stratified by duration of diabetes (< 10 and ≥ 10 years), hypertension status, and statin use.

We used sampling weights to obtain nationally representative estimates of the non-institutionalized US population with diabetes. Analyses accounted for the multi-stage selection of study participants and were conducted using SAS 9.4 (SAS Institute, Cary, NC). *P*-values < 0.05 were considered statistically significant.

## RESULTS

### Participant Characteristics

**Figure 1:**
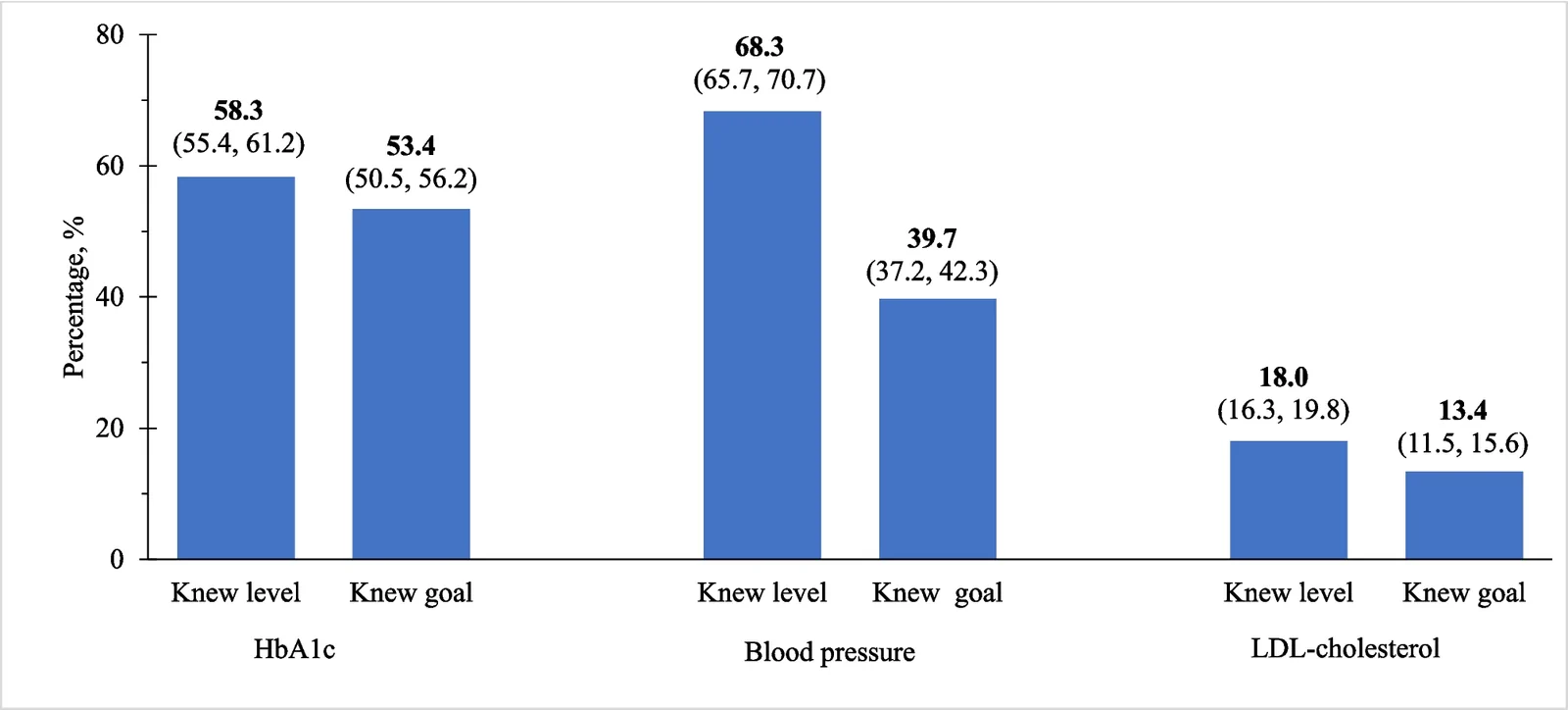
Percentage (95% confidence interval) of US adults with diabetes who reported knowing their recent HbA1c, blood pressure, and LDL-cholesterol levels and goals. The numbers in the figure are percentage (95% confidence interval). We defined reported knowledge of blood pressure levels and goals based on having a response for both systolic and diastolic blood pressure. HbA1c: glycated hemoglobin A1c; LDL: low-density lipoprotein.

The mean age of US adults with diabetes was 60.4 years, 52.7% were men, and 59.8% self-identified as non-Hispanic White (Table 1). Overall, 58.3%, 68.3 and 18.0% US adults with diabetes reported that they knew their recent HbA1c, BP, and LDL-cholesterol levels, respectively, and 53.4%, 39.7%, and 13.4% reported they knew their HbA1c, BP, and LDL-cholesterol goals as recommended by their doctor or health care provider, respectively (Fig. 1). The distributions of self-reported HbA1c, BP, and LDL-cholesterol levels and goals among participants with diabetes included in the analysis are presented in Supplemental Fig. 2.

**Table 1:** Characteristics of US Adults with Diabetes – National Health and Nutrition Examination Surveys 2011–2020

Characteristics	Percentage or mean (95% confidence interval) *N* = 3,464
Sociodemographic	
Mean age, years	60.4 (59.8, 61.1)
Age group, years, %	
18 to 49	20.2 (18.4, 22.0)
50 to 59	23.8 (21.0, 26.7)
60 to 69	27.8 (25.2, 30.5)
≥ 70	28.3 (26.3, 30.4)
Male sex, %	52.7 (50.1, 55.3)
Race/Ethnicity, %	
Non-Hispanic White	59.8 (55.9, 63.7)
Non-Hispanic Black	14.2 (11.7, 17.0)
Hispanic	15.9 (13.2, 19.0)
Other	10.0 (8.4, 11.7)
Less than high school education, %	20.4 (18.2, 22.8)
Health care access, %	
Had health insurance coverage	91.0 (89.7, 92.3)
Cardiovascular disease risk factors	
History of cardiovascular disease, %	9.4 (7.9, 10.9)
Chronic kidney disease, %	17.1 (15.6, 18.6)
Hypertension, %*	69.4 (66.9, 71.8)
Mean systolic BP, mm Hg	130.0 (129.0, 131.0)
Mean diastolic BP, mm Hg	70.6 (70.0, 71.2)
Diabetes diagnosis, ≥ 10 years, %	50.1 (47.4, 52.9)
Laboratory measures	
Mean HbA1c, %	7.4 (7.3, 7.5)
HbA1c ≥ 7%, %	48.6 (45.5, 51.7)
Mean LDL-cholesterol, mg/dL^†^	102.0 (99.0, 104.9)
LDL-cholesterol ≥ 100 mg/dL,^†^ %	45.9 (41.5, 50.4)
Medication use	
Antihypertensive medication use, %	63.1 (60.3, 65.8)
Statin use, %	56.6 (53.7, 59.5)

*BP* Blood pressure, *CI* Confidence interval, *HbA1c* glycated hemoglobin A1c, *LDL* Low-density lipoprotein

^†^Analytic population includes only participants in the fasting subsample, *N* = 950

The numbers in the table are weighted percentage (95% confidence interval) or mean (95% confidence interval) of the specified subgroup of US population with diabetes

*Hypertension was defined as systolic blood pressure of ≥ 140 or diastolic blood pressure ≥ 90 mmHg or taking antihypertensive medication

### Association of Participant Characteristics with Reported Knowledge of HbA1c, BP, and LDL-Cholesterol Levels and Goals

Adults 60 years and older versus 18–59 years of age, male versus female, Non-Hispanic White versus Non-Hispanic Black or Hispanic participants were more likely to report knowing their recent HbA1c, BP and LDL-cholesterol levels and goals (Table 2). Participants with less than high school education versus greater than high school education and those without versus with health insurance were less likely to report knowing their recent HbA1c, BP, and LDL-cholesterol levels and goals. After adjustment for sex, US adults 60 to 69 and ≥ 70 years of age versus 18 to 49 years of age were more likely to report knowing their recent BP and LDL-cholesterol levels and LDL-cholesterol goals (Table 3). After age and sex adjustment, participants who identified as non-Hispanic Black or Hispanic versus non-Hispanic White, and participants with less than high school education versus high school education or above, and without versus with health insurance were less likely to report knowing their HbA1c, BP and LDL-cholesterol levels and goals. Participants who had versus did not have hypertension and participants who were taking versus not taking antihypertensive medication were more likely to report knowing their recent BP levels. Participants who had diabetes for ≥ 10 years versus < 10 years and who were taking versus not taking a statin were more likely to report knowing their recent HbA1c levels and goals.

**Table 2:** Percentage (95% Confidence interval) of US Adults with Diabetes Who Reported Knowing Their HbA1c, Blood Pressure, and LDL-Cholesterol Values and Goals

	HbA1c		Systolic and diastolic BP		LDL-cholesterol	
**Characteristics**	Knew value	Knew goal	Knew value	Knew goal	Knew value	Knew goal
Sociodemographic						
Age group, years						
18 to 49	56.7 (51.1, 62.1)	53.5 (47.8, 59.1)	57.9 (51.5, 64.2)	36.4 (29.8, 43.4)	10.4 (7.5, 13.8)	6.6 (4.0, 10.2)
50 to 59	59.5 (53.1, 65.6)	57.5 (50.9, 63.9)	66.4 (60.1, 72.2)	41.6 (35.2, 48.2)	18.4 (13.6, 23.9)	12.5 (8.3, 17.8)
60 to 69	61.3 (55.9, 66.6)	54.7 (49.0, 60.3)	74.1 (70.1, 77.9)	41.8 (36.1, 47.6)	20.7 (16.5, 25.5)	18.8 (14.8, 23.4)
≥ 70	55.6 (50.8, 60.3)	48.5 (43.7, 53.2)	71.5 (68.0, 74.9)	38.5 (33.9, 43.2)	20.5 (17.0, 24.4)	13.9 (9.8, 18.9)
Sex						
Female	56.4 (52.1, 60.6)	51.1 (46.8, 55.4)	67.8 (64.6, 70.9)	38.0 (34.8, 41.2)	15.5 (12.9, 18.3)	12.6 (10.2, 15.3)
Male	60.0 (56.6, 63.4)	55.4 (51.9, 58.8)	68.7 (64.8, 72.5)	41.3 (37.3, 45.3)	20.3 (17.4, 23.4)	14.2 (11.4, 17.4)
Race/Ethnicity						
Non-Hispanic White	66.7 (63.3, 69.9)	61.2 (57.9, 64.4)	74.6 (71.4, 77.7)	42.7 (38.7, 46.6)	21.9 (18.8, 25.2)	17.0 (13.6, 20.9)
Non-Hispanic Black	44.6 (40.4, 48.8)	41.2 (36.8, 45.7)	61.5 (57.6, 65.3)	37.2 (33.8, 40.7)	6.8 (5.1, 8.9)	4.9 (3.4, 6.9)
Hispanic	37.5 (33.5, 41.6)	34.6 (30.4, 39.0)	48.1 (44.1, 52.2)	31.1 (26.9, 35.6)	10.7 (8.3, 13.4)	7.1 (5.3, 9.3)
Other	61.0 (54.4, 67.4)	53.7 (46.9, 60.3)	71.9 (66.0, 77.3)	39.4 (33.2, 45.8)	22.6 (15.7, 30.8)	14.3 (9.4, 20.5)
Education						
High school or above	65.3 (62.1, 68.4)	60.0 (56.9, 63.0)	73.2 (70.3, 75.9)	43.3 (40.2, 46.6)	20.1 (17.9, 22.5)	15.2 (12.7, 18.1)
Less than high school	31.2 (26.8, 35.8)	27.5 (23.1, 32.2)	49.6 (45.4, 53.8)	26.0 (22.6, 29.6)	10.1 (7.3, 13.5)	6.6 (4.3, 9.6)
Health care access						
Had health insurance coverage						
No	34.1 (27.7, 40.9)	30.7 (24.3, 37.7)	51.9 (45.4, 58.3)	28.4 (22.6, 34.8)	8.5 (4.9, 13.5)	5.9 (3.2, 9.8)
Yes	60.7 (57.7, 63.7)	55.6 (52.7, 58.5)	69.9 (67.2, 72.5)	40.8 (38.2, 43.5)	19.0 (17.1, 20.9)	14.2 (12.0, 16.6)
Cardiovascular disease risk factors						
History of cardiovascular disease						
No	59.1 (56.2, 61.9)	53.9 (51.0, 56.7)	68.0 (65.3, 70.5)	39.4 (36.7, 42.1)	18.3 (16.4, 20.4)	13.9 (11.6, 16.4)
Yes	51.0 (42.5, 59.5)	48.0 (39.0, 57.0)	71.9 (64.9, 78.2)	44.0 (36.0, 52.2)	15.2 (8.8, 23.8)	9.3 (5.2, 15.0)
Chronic kidney disease						
No	58.8 (55.3, 62.3)	54.3 (50.9, 57.8)	67.5 (64.4, 70.5)	39.6 (36.5, 42.7)	18.4 (16.5, 20.4)	13.7 (11.4, 16.4)
Yes	56.2 (51.1, 61.3)	49.3 (44.1, 54.5)	71.5 (66.4, 76.3)	40.5 (34.7, 46.5)	16.4 (12.8, 20.7)	12.3 (8.9, 16.5)
Hypertension, BP ≥ 140/90 mm Hg						
No	54.3 (49.7, 58.9)	50.9 (46.5, 55.3)	56.8 (52.2, 61.3)	27.6 (23.6, 31.9)	17.7 (14.5, 21.1)	9.9 (7.2, 13.1)
Yes	60.1 (56.2, 63.8)	54.4 (50.4, 58.4)	73.4 (70.5, 76.0)	45.1 (41.7, 48.4)	18.2 (15.8, 20.7)	15.0 (12.5, 17.9)
Diabetes diagnosis, ≥ 10 years						
No	52.7 (47.5, 57.9)	47.9 (42.8, 53.0)	67.8 (64.0, 71.4)	38.8 (34.8, 43.0)	16.7 (13.9, 19.8)	11.4 (8.9, 14.3)
Yes	64.3 (61.1, 67.4)	59.3 (56.1, 62.3)	69.3 (66.1, 72.4)	41.1 (37.1, 45.1)	19.5 (16.4, 23.0)	15.6 (12.2, 19.5)
Laboratory measures						
HbA1c level ≥ 7%						
No	55.0 (51.7, 58.2)	49.3 (46.1, 52.5)	70.2 (66.8, 73.4)	40.5 (36.8, 44.4)	20.3 (17.6, 23.2)	14.5 (11.7, 17.8)
Yes	62.2 (58.4, 65.9)	57.9 (53.9, 61.7)	66.3 (62.5, 69.9)	39.0 (35.7, 42.3)	15.7 (13.3, 18.3)	12.1 (10.0, 14.6)
LDL-cholesterol ≥ 100 mg/dL^†^						
No	62.6 (55.9, 69.1)	56.5 (48.7, 64.0)	67.8 (60.9, 74.2)	40.2 (34.6, 46.0)	17.6 (12.4, 23.8)	12.4 (7.6, 18.8)
Yes	51.5 (44.7, 58.2)	47.4 (40.0, 54.8)	67.9 (61.8, 73.6)	38.8 (31.8, 46.3)	17.1 (12.3, 22.7)	15.4 (10.1, 22.1)
Medication use						
Antihypertensive medication use						
No	54.9 (50.9, 58.8)	51.0 (47.2, 54.8)	56.9 (52.7, 61.0)	28.5 (24.6, 32.6)	17.5 (14.7, 20.7)	10.3 (7.8, 13.2)
Yes	61.2 (57.1, 65.2)	55.4 (51.1, 59.6)	75.7 (72.8, 78.4)	47.0 (43.3, 50.8)	18.6 (16.2, 21.2)	15.5 (12.9, 18.5)
Statin use						
No	54.3 (50.3, 58.1)	49.8 (46.0, 53.6)	62.6 (59.0, 66.1)	36.4 (32.4, 40.5)	14.0 (11.9, 16.4)	10.1 (7.5, 13.3)
Yes	61.4 (57.1, 65.6)	56.1 (51.5, 60.6)	72.7 (69.1, 76.1)	42.2 (38.1, 46.4)	21.1 (18.3, 24.2)	15.9 (13.0, 19.1)

The numbers in the table are weighted row percentage (95% confidence interval)

*BP* Blood pressure, *HbA1c* glycated hemoglobin A1c, *LDL* Low-density lipoprotein

Hypertension was defined as systolic blood pressure of ≥ 140 or diastolic blood pressure ≥ 90 mmHg or taking antihypertensive medication

^†^Analytic population includes only participants in the fasting subsample, *N *= 950

### Association Between Reported Knowledge of Recent Levels and Goals with Risk Factor Control

The mean (95% CI) HbA1c levels for participants who reported not knowing and knowing their recent HbA1c level was 7.3 (95% CI: 7.2, 7.4) and 7.4 (95% CI: 7.3, 7.5), respectively, and not knowing and knowing their HbA1c goal was 7.3 (95%CI: 7.2, 7.4), 7.5 (95% CI: 7.3, 7.6), respectively (Supplemental Table 2). Among participants who reported knowing their recent HbA1c levels and goals, 48.3% (95% CI: 44.5, 52.2) and 47.4% (95% CI: 43.3, 51.6) had controlled HbA1c, respectively. After age and sex adjustment, US adults with diabetes who reported knowing versus not knowing their recent HbA1c levels and goals were less likely to have controlled HbA1c (PR: 0.86; 95% CI: 0.79, 0.93 and PR: 0.86; 95% CI: 0.80, 0.94, respectively). After age and sex adjustment, mean diastolic BP was 1.41 (95% CI 0.26, 2.56) mm Hg higher for those knowing versus not knowing their recent diastolic BP. There was no evidence of an association between reporting knowledge of systolic or diastolic BP levels or goals with BP control. Reported knowledge of recent LDL-cholesterol levels and goals were not associated with higher LDL-cholesterol levels or LDL-cholesterol control.

**Table 3:** Prevalence ratios (95% Confidence interval) for Knowing Recent HbA1c, Blood Pressure, LDL-Cholesterol Levels, and Their Respective Goals Among US Adults With Diabetes

	HbA1c		Systolic and diastolic BP		LDL-cholesterol	
	Knew value	Knew goal	Knew value	Knew goal	Knew value	Knew goal
Sociodemographic						
Age group, years						
18 to 49	1 (ref)	1 (ref)	1 (ref)	1 (ref)	1 (ref)	1 (ref)
50 to 59	1.05 (0.90, 1.21)	1.07 (0.91, 1.26)	1.14 (0.98, 1.33)	1.14 (0.87, 1.49)	**1.75 (1.12, 2.73)**	1.89 (1.00, 3.57)
60 to 69	1.08 (0.93, 1.24)	1.02 (0.87, 1.19)	**1.28 (1.13, 1.45)**	1.14 (0.92, 1.41)	**1.96 (1.37, 2.81)**	**2.83 (1.69, 4.74)**
≥ 70	0.98 (0.87, 1.10)	0.91 (0.79, 1.04)	**1.23 (1.09, 1.40)**	1.06 (0.84, 1.33)	**1.97 (1.34, 2.89)**	**2.10 (1.17, 3.77)**
Sex						
Female	1 (ref)	1 (ref)	1 (ref)	1 (ref)	1 (ref)	1 (ref)
Male	1.06 (0.97, 1.15)	1.08 (0.98, 1.19)	1.01 (0.94, 1.08)	1.08 (0.95, 1.23)	**1.29 (1.01, 1.66)**	1.10 (0.85, 1.42)
Race/Ethnicity						
Non-Hispanic White	1 (ref)	1 (ref)	1 (ref)	1 (ref)	1 (ref)	1 (ref)
Non-Hispanic Black	**0.66 (0.60, 0.73)**	**0.67 (0.60, 0.74)**	**0.83 (0.77, 0.90)**	0.88 (0.77, 1.00**)**	**0.33 (0.24, 0.45)**	**0.30 (0.20, 0.45)**
Hispanic	**0.55 (0.49, 0.62)**	**0.55 (0.48, 0.63)**	**0.66 (0.60, 0.73)**	**0.73 (0.62, 0.87)**	**0.54 (0.40, 0.71)**	**0.46 (0.32, 0.66)**
Other	0.91 (0.81, 1.01)	0.87 (0.76, 0.99)	0.97 (0.89, 1.05)	0.92 (0.77, 1.10)	1.05 (0.72, 1.54)	0.84 (0.55, 1.29)
Education						
High school or above	1 (ref)	1 (ref)	1 (ref)	1 (ref)	1 (ref)	1 (ref)
Less than high school	**0.48 (0.42, 0.55)**	**0.46 (0.39, 0.55)**	**0.67 (0.62, 0.74)**	**0.60 (0.52, 0.71)**	**0.50 (0.36, 0.71)**	**0.43 (0.27, 0.68)**
Health care access						
Health insurance coverage						
Yes	1 (ref)	1 (ref)	1 (ref)	1 (ref)	1 (ref)	1 (ref)
No	**0.55 (0.45, 0.67)**	**0.53 (0.42, 0.65)**	**0.78 (0.68, 0.89)**	**0.70 (0.56, 0.86)**	**0.51 (0.31, 0.86)**	**0.49 (0.27, 0.88)**
Cardiovascular disease risk factors						
History of cardiovascular disease						
No	1 (ref)	1 (ref)	1 (ref)	1 (ref)	1 (ref)	1 (ref)
Yes	0.86 (0.74, 1.00)	0.91 (0.76, 1.08)	1.02 (0.93,1.13)	1.11 (0.93, 1.33)	0.75 (0.45, 1.26)	0.60 (0.33, 1.07)
Chronic kidney disease						
No	1 (ref)	1 (ref)	1 (ref)	1 (ref)	1 (ref)	1 (ref)
Yes	0.97 (0.86, 1.09)	0.95 (0.82, 1.10)	1.01 (0.93, 1.09)	1.03 (0.86, 1.23)	0.77 (0.59, 1.01)	0.77 (0.51, 1.15)
Hypertension, ≥140/90 mmHg						
No	1 (ref)	1 (ref)	1 (ref)	1 (ref)	1 (ref)	1 (ref)
Yes	1.11 (1.00, 1.24)	1.09 (0.97, 1.23)	**1.25 (1.16, 1.34)**	**1.67 (1.41, 1.97)**	0.89 (0.68, 1.15)	1.28 (0.89, 1.85)
Duration of diabetes diagnosis						
<10 years	1 (ref)	1 (ref)	1 (ref)	1 (ref)	1 (ref)	1 (ref)
≥10 years	**1.24 (1.11, 1.39)**	**1.29 (1.14, 1.45)**	0.98 (0.92, 1.06)	1.06 (0.91,1.24)	1.07 (0.82, 1.41)	1.26 (0.91, 1.76)
Laboratory measures						
HbA1c level						
<7%	1 (ref)	1 (ref)	1 (ref)	1 (ref)	1 (ref)	1 (ref)
≥7%	**1.13 (1.06, 1.20)**	**1.16 (1.07, 1.25)**	0.95 (0.89, 1.02)	0.95 (0.84, 1.08)	**0.76 (0.61, 0.95)**	0.84 (0.66, 1.09)
LDL-cholesterol ≥100 mg/dL^†^						
No	1 (ref)	1 (ref)	1 (ref)	1 (ref)	1 (ref)	1 (ref)
Yes	**0.80 (0.70, 0.92)**	**0.80 (0.67, 0.95)**	1.02 (0.90, 1.15)	0.95 (0.78, 1.15)	1.03 (0.67, 1.59)	1.29 (0.75, 2.20)
Medication use						
Antihypertensive medication use						
No	1 (ref)	1 (ref)	1 (ref)	1 (ref)	1 (ref)	1 (ref)
Yes	**1.12 (1.02, 1.24)**	1.11 (1.00, 1.24)	**1.30 (1.20, 1.40)**	**1.69 (1.45, 1.98)**	0.94 (0.76, 1.18)	1.31 (0.97, 1.76)
Statin use						
No	1 (ref)	1 (ref)	1 (ref)	1 (ref)	1 (ref)	1 (ref)
Yes	**1.14 (1.04, 1.25)**	**1.15 (1.03, 1.28)**	**1.11 (1.03, 1.20)**	1.14 (0.96, 1.37)	**1.32 (1.04, 1.66)**	1.34 (0.92, 1.97)

The numbers in the table are prevalence ratios (95% confidence interval). The prevalence ratios were calculated adjusting for age and sex (except for age which was adjusted for sex and sex which was adjusted for age)

*BP* Blood pressure, *HbA1c* glycated hemoglobin A1c, *LDL* Low-density lipoprotein

^†^Analytic population includes only participants in the fasting subsample, *N* = 950 Hypertension was defined as systolic blood pressure of ≥ 140 or diastolic blood pressure ≥ 90 mmHg or taking antihypertensive medication

### Stratified Analyses

There was no evidence of an association between reported knowledge of recent HbA1c levels or goals and HbA1c control for those with diabetes for < 10 or ≥ 10 years (Supplemental Table 3). Among US adults with diabetes and hypertension, SBP was lower among those who reported knowing versus not knowing their recent systolic BP (Supplemental Table 4). BP control was higher among those with diabetes and hypertension who reported knowing versus not knowing their recent systolic and diastolic BP levels. Among US adults with diabetes who were not and who were taking a statin, reported knowledge of recent LDL-cholesterol levels was associated with higher mean LDL-cholesterol. Among those taking a statin, reported knowledge of LDL-cholesterol goals was also associated with higher mean LDL-cholesterol (Supplemental Table 5).

## DISCUSSION

In the current study, a low percentage of US adults with diabetes reported knowing their HbA1c, BP, or LDL-cholesterol levels and respective goals as recommended by their doctor or health care professional. Non-Hispanic Black and Hispanic versus non-Hispanic White adults, those with less than high school education versus those with a high school education or higher, and those without health insurance versus those with health insurance were less likely to report knowing their recent HbA1c, BP, or LDL-cholesterol levels and goals.

Prior studies among adults with diabetes have found a low percentage of individuals knew their HbA1c level and goal.^7,8^ In a study by Heisler et al. using data from 5 health systems, only 34% of adults with diabetes knew their last HbA1c value and only 25% reported knowing their HbA1c value correctly.^7^ In NHANES 2005–2008, 48% of US adults with diabetes reported knowing their HbA1c level.^9^ The current study documents an increase in the proportion of adults with diabetes who report knowing their recent HbA1c levels and goals. In the current study, US adults with diabetes who reported knowing their HbA1c level were less likely to have controlled HbA1c compared to their counterparts who reported not knowing their HbA1c level or goal. As ADA Standards of Care recommend more frequent HbA1c monitoring among individuals whose HbA1c is uncontrolled,^14^ individuals with HbA1c above goal may report greater awareness than those with controlled HbA1c. However, the gaps in HbA1c control among those who report knowing their HbA1c levels or goals reinforce that knowledge of HbA1c is important but may not be sufficient for improving diabetes management.^7^

In NHANES 2005–2008, 63% of adults with diabetes reported knowing their BP level, however only 26.7% reported knowing their doctor or health care provider recommended BP goal.^9^ While the results from the current study indicate there has been an increase in the proportion of adults with diabetes who report knowing their recent BP levels and goals, the percentage who reported knowing their recommended BP goal remains low.^8^ During the NHANES cycles included in this analysis (i.e., 2011 through March 2020), multiple professional societies updated BP thresholds for defining hypertension and BP treatment goals for adults with diabetes, and these changes might have led to inconsistent BP goals clinicians communicated and created a potential confusion among healthcare providers and patients about BP goals.^5,6,12^ Not receiving a hypertension diagnosis and not being aware of having high BP among adults with diabetes results in uncontrolled BP and potential target organ damage.^15^ Therefore, additional efforts are needed to diagnose and manage hypertension among adults with diabetes to reduce the risk of CVD and microvascular complications.^16,17^

In the current study, US adults with diabetes were less likely to report knowledge of LDL-cholesterol levels and goals compared to knowledge of HbA1c levels and goals. Among adults aged 40 to 75 years with diabetes, ADA Standards of Care recommend treatment with a moderate intensity statin if no additional risk factors are present and treatment with a high intensity statin with an LDL-cholesterol goal of < 70 mg/dL or < 55 mg/dL for those with atherosclerotic CVD (ASCVD) risk factors or ASCVD, respectively.^5^ Prior analyses of NHANES data show that the proportion of adults with diabetes with LDL cholesterol at goal plateaued in recent years, and a lower proportion of adults with diabetes report knowing LDL-cholesterol goals compared to HbA1c or BP goals.^4,9^ In the current study, reported knowledge of LDL-cholesterol was not associated with better LDL-cholesterol control, and among statin users, reported knowledge was associated with higher LDL-cholesterol levels. This is likely because individuals with high LDL-cholesterol levels may receive more intensive counseling and monitoring for lipid control, as opposed to a causal relationship between knowledge and higher LDL-cholesterol levels.^16^ Understanding reasons for the low knowledge of LDL-cholesterol levels and goals may inform strategies to increase awareness and support diabetes self-management behaviors.

A prior study has reported that knowledge of HbA1c, BP and LDL-cholesterol was higher in Non-Hispanic White individuals compared to Non-Hispanic Black or Hispanic individuals, and those with higher education and income.^9^ In the current study, participants who identified as Non-Hispanic Black or Hispanic, who had less than high school education, and those without health insurance were less likely to report knowing their recent HbA1c, BP, or LDL-cholesterol levels and goals. As the prevalence of diabetes is higher among these population subgroups, interventions are needed to address the factors that contribute to the development of diabetes and poorer control of cardiovascular risk factors in this population. Achieving health equity will require interventions to address structural racism and downstream consequences on the neighborhood built and social environments.^18,19^ Simultaneously, interventions that increase access to care, identify adverse social determinants of health and link patients to community resources, promote health literacy, and deliver patient-centered care, such as by incorporating community health workers into diabetes care, may help overcome barriers to diabetes management.^20^

Clinical and public health interventions are needed to increase knowledge of risk factor levels and goals among adults with diabetes, and support improved cardiovascular risk factor management. For example, health care and public health organizations could expand availability and access to DSMES. Individuals with diabetes are recommended DSMES at various times throughout their disease course at diagnosis, annually or when not meeting treatment targets, when complicating factors develop, or with transitions in care.^21,22^ DSMES is delivered by Diabetes Care and Education Specialists from a range of health care disciplines and imparts knowledge, decision-making, and skills necessary for optimal diabetes self-care through a person-centered approach.^22^ DSMES helps adults with diabetes to improve self-management behaviors and control risk factor levels, including addressing barriers to their implementation, including social determinants of health.^23–25^ Thus, DSMES is designed not only to increase knowledge but to address barriers to self-management of diabetes, which may be necessary to improve risk factor control. However, DSMES remains underutilized due to factors such as low referral rates, programmatic capacity, and barriers to participation.^22^ Health systems strategies including electronic dashboards, automated reminders, and structured communication protocols may help increase patient awareness of risk factor levels and goals.^26^ In addition, clinician-level strategies including visual aids, and numeracy-focused education may also support patients to interpret and act on their risk factor information on HbA1c, BP, and LDL-cholesterol levels and achieve their risk factor goals.^26,27^ It may be necessary to culturally tailor programmatic elements to meet the needs of and increase uptake among racial and ethnic minorities and other underserved population groups.^28,29^

The current study has several strengths. NHANES participants are selected such that results can be weighted to provide nationally representative data for non-institutionalized US adults. BP was measured following standardized procedures, and HbA1c and cholesterol values were measured using rigorous procedures. There are potential and known limitations. Due to a relatively small sample size in certain subgroups, particularly in the fasting subsample, we may not have been able to detect the associations of risk factors with reported knowledge of LDL-cholesterol levels and goals. We relied on self-reported responses to determine awareness of HbA1c, BP, and LDL-cholesterol levels and goals rather than numeric accuracy of these responses. Therefore, the results should be interpreted as indicators of perceived knowledge. However, we could not determine if participants were told their risk factor levels and goals or not. It is possible that participants had been told their HbA1c, BP and LDL-cholesterol levels and goals and forgot but it is possible they had not been told. Participants’ reported knowledge of numeric values may represent recall of clinician communication and may not necessarily convey understanding of the clinical meaning or implications of these values but serves as an indicator of clinician-patient engagement. Determining reasons participants did not know their HbA1c, BP and LDL-cholesterol levels and goals may facilitate interventions to increase risk factor awareness and effective self-management.

In conclusion, a low percentage of US adults with diabetes reported knowing their HbA1c, BP, or LDL-cholesterol levels and goals. Disparities were present in the reported knowledge of HbA1c, BP, and LDL-cholesterol levels and goals with lower percentages among non-Hispanic Black and Hispanic vs non-Hispanic White adults, less than high school vs high school or above education, and those without vs with health insurance. These data indicate gaps in reported knowledge of HbA1c, BP, and LDL-cholesterol levels and goals among US adults with diabetes.

## Supplementary Information

Below is the link to the electronic supplementary material.

ESM 1 (DOCX 99.9 KB)

## Data Availability

Data analyzed in the current study are publicly available from the CDC at https://wwwn.cdc.gov/nchs/nhanes/default.aspx.
