# Supplementary material for: Reported Knowledge of Recent HbA1c, Blood Pressure, and LDL-cholesterol Levels and Goals Among US Adults with Diabetes—National Health and Nutrition Examination Survey 2011–2020
Source: J Gen Intern Med. 2026 May 6;41(14):4046–54. doi: 10.1007/s11606-026-10474-8 (PMC13728023; doi:10.1007/s11606-026-10474-8)
Supplement: Supplementary file 1 [file 11606_2026_10474_MOESM1_ESM.docx]

**Reported knowledge of recent HbA1c, blood pressure, and LDL-cholesterol levels and goals among US adults with diabetes - National Health And Nutrition Examination Survey 2011-2020**

**Short title**: Knowledge of HbA1c, BP, and LDL-C in diabetes.

Bharat Poudel, MSPH^1^; Ligong Chen, PhD^2^; Medha J Dubal, MPH^1^; Ashley E Christenson, BS^1^; Caroline Presley, MD^1^; Paul Muntner, PhD^2^; Michael Fang, PhD^3^; Kathryn Foti, PhD^1^

^1^University of Alabama at Birmingham, AL, USA; ^2^Perisphere Real World Evidence LLC, Austin, TX, USA; ^3^Johns Hopkins Bloomberg School of Public Health, Baltimore, MD, USA.

**Supplemental Materials**:

Supplemental Figure 1. Flow-chart showing the number of NHANES (2011 to 2017-2020) participants included in the current analyses.

Completed NHANES interview and examination

N= 42,295

Participants who are at least 18 years of age

N= 26,239

Participants who had diabetes*

N= 3,518

Participants who are not pregnant

N=25,960

Participants who had information on outcome data:

Knew HbA1C level and goal

N=3,501

Knew recent systolic and diastolic blood pressure value and goal

N=3,480

Knew LDL-cholesterol value and goal

N=3,464

*Diabetes is defined as a self-reported history of diabetes.

Supplemental Figure 2. Distribution of HbA1c (top panel), blood pressure (middle panel), and low-density lipoprotein cholesterol (bottom panel) levels and goals among NHANES participants with diabetes.

Distribution of last measured HbA1c level

| HbA1c, % | Percentage, % |
| --- | --- |
| < 5.7 | 8.1 |
| 5.7 to <6.5 | 21.5 |
| 6.5 to <7.5 | 38.3 |
| 7.5 to <8.5 | 15.1 |
| 8.5 to <10 | 8.3 |
| >=10 | 8.8 |

Distribution of HbA1c goal

| HbA1c, % | Percentage, % |
| --- | --- |
| < 6.0 | 48.3 |
| <7.0 | 42.5 |
| <8.0 | 6.3 |
| <9.0 | 1.2 |
| <10.0 | 1.7 |

Distribution of recent blood pressure level

| Blood pressure, mm Hg | Percentage, % |
| --- | --- |
| <120/80 | 21.9 |
| 120-140/80-90 | 33 |
| >=140/90 | 45.1 |

Distribution of blood pressure goal

| Blood pressure, mm Hg | Percentage, % |
| --- | --- |
| <120/80 | 8.2 |
| 120-140/80-90 | 74 |
| >=140/90 | 17.7 |

Distribution of LDL-cholesterol level

| LDL-cholesterol, mg/dL | Percentage, % |
| --- | --- |
| <100 | 40.3 |
| 100-130 | 17.1 |
| 130-160 | 15.2 |
| 160-190 | 11.5 |
| >=190 | 15.8 |

Distribution of LDL-cholesterol goal

| LDL-cholesterol, mg/dL | Percentage, % |
| --- | --- |
| <100 | 32.2 |
| 100-130 | 31 |
| 130-160 | 17 |
| 160-190 | 7.3 |
| >=190 | 12.6 |

Supplemental Table 1. Definition of levels and goals for HbA1c, blood pressure, and low-density lipoprotein cholesterol.

| Outcome variables | NHANES cycles | | | |
| --- | --- | --- | --- | --- |
|  | 2011-2012 | 2013-2014 | 2015-2016 | 2017-March 2020 |
| HbA1c level | What was your last A1C level? | | | |
|  | 2.1 to 36 \| Range of values; 777 \| Refused; 999 \| Don’t know; . \| Missing | 4.1 to 14 \| Range of values; 777 \| Refused; 999 \| Don’t know; . \| Missing | 2.0 to 18.5 \| Range of values; 777 \| Refused; 999 \| Don’t know; . \| Missing | 2.3 to 16.0 \| Range of values; 777 \| Refused; 999 \| Don’t know; . \| Missing |
| HbA1c goal | What does Dr say A1C should be? 1 \| <6; 2 \| <7; 3 \| <8; 4 \| <9; 5 \| <10; 6 \| Provider did not specify goal; 77 \| Refused; 99 \| Don’t know; . \| Missing | | | |
| SBP level | What was your recent SBP? | | | |
|  | 63 to 220 \| Range of values; 7777 \| Refused; 9999 \| Don’t know; . \| Missing | 88 to 255 \| Range of values; 7777 \| Refused; 9999 \| Don’t know; . \| Missing | 80 to 201 \| Range of values; 7777 \| Refused; 9999 \| Don’t know; . \| Missing | 58 to 212 \| Range of values; 7777 \| Refused; 9999 \| Don’t know; . \| Missing |
| DBP level | What was your recent DBP? | | | |
|  | 25 to 250 \| Range of values; 7777 \| Refused; 9999 \| Don’t know; . \| Missing | 5 to 175 \| Range of values; 7777 \| Refused; 9999 \| Don’t know; . \| Missing | 17 to 251 \| Range of values; 7777 \| Refused; 9999 \| Don’t know; . \| Missing | 25 to 190 \| Range of values; 7777 \| Refused; 9999 \| Don’t know; . \| Missing |
| SBP goal | What does Dr say SBP should be? | | | |
|  | 70 to 190 \| Range of values; 6666 \| Provider did not specify goal; 7777 \| Refused; 9999 \| Don’t know; . \| Missing | 70 to 180 \| Range of values; 6666 \| Provider did not specify goal; 7777 \| Refused; 9999 \| Don’t know; . \| Missing | 80 to 175 \| Range of values; 6666 \| Provider did not specify goal; 7777 \| Refused; 9999 \| Don’t know; . \| Missing | 72 to 180 \| Range of values; 6666 \| Provider did not specify goal; 7777 \| Refused; 9999 \| Don’t know; . \| Missing |
| DBP goal | What does Dr say DBP should be? | | | |
|  | 20 to 140 \| Range of values; 6666 \| Provider did not specify goal; 7777 \| Refused; 9999 \| Don’t know; . \| Missing | 5 to 120 \| Range of values; 6666 \| Provider did not specify goal; 7777 \| Refused; 9999 \| Don’t know; . \| Missing | 18 to 140 \| Range of values; 6666 \| Provider did not specify goal; 7777 \| Refused; 9999 \| Don’t know; . \| Missing | 20 to 140 \| Range of values; 6666 \| Provider did not specify goal; 7777 \| Refused; 9999 \| Don’t know; . \| Missing |
| LDL-cholesterol level | What was most recent LDL number? | | | |
|  | 18 to 300 \| Range of values; 5555 \| Never heard of LDL; 6666 \| Never had cholesterol test; 7777 \| Refused; 9999 \| Don’t know; . \| Missing | 5 to 350 \| Range of values; 5555 \| Never heard of LDL; 6666 \| Never had cholesterol test; 7777 \| Refused; 9999 \| Don’t know; . \| Missing | 4 to 520 \| Range of values; 5555 \| Never heard of LDL; 6666 \| Never had cholesterol test; 7777 \| Refused; 9999 \| Don’t know; . \| Missing | 8 to 340 \| Range of values; 5555 \| Never heard of LDL; 6666 \| Never had cholesterol test; 7777 \| Refused; 9999 \| Don’t know; . \| Missing |
|  | What does Dr say LDL should be? | | | |
| LDL-cholesterol goal | 7 to 268 \| Range of values; 6666 \| Provider did not specify goal; 7777 \| Refused; 9999 \| Don’t know; . \| Missing | 2 to 250 \| Range of values; 6666 \| Provider did not specify goal; 7777 \| Refused; 9999 \| Don’t know; . \| Missing | 6 to 205 \| Range of values; 6666 \| Provider did not specify goal; 7777 \| Refused; 9999 \| Don’t know; . \| Missing | 15 to 250 \| Range of values; 6666 \| Provider did not specify goal; 7777 \| Refused; 9999 \| Don’t know; . \| Missing |

The range of values for HbA1c, BP, and LDL-cholesterol represented the reported knowledge of HbA1c, systolic and diastolic BP, and LDL-cholesterol levels and goals.

SBP: systolic blood pressure, DBP: diastolic blood pressure; LDL: low-density lipoprotein.

Supplemental Table 2. Mean (95% confidence interval) and percentage control for reported knowledge of recent HbA1c, blood pressure, LDL-cholesterol levels and goals and their associations with mean levels and control of HbA1c, blood pressure, and LDL-cholesterol, among US adults with diabetes.

|  | Mean level (95% CI) | Adjusted difference* (95% CI) | Percentage with control  (95% CI) | Adjusted prevalence ratio for control* (95% CI) |
| --- | --- | --- | --- | --- |
|  | **HbA1c** | | | |
| HbA1c, % | 7.4 (7.3, 7.5) |  | 51.4 (48.3, 54.5) |  |
| Knew recent HbA1c level |  |  |  |  |
| No | 7.3 (7.2, 7.4) | 0 (ref) | 55.8 (52.3, 59.2) | 1 (ref) |
| Yes | 7.4 (7.3, 7.5) | 0.09 (-0.03, 0.21) | 48.3 (44.5, 52.2) | **0.86 (0.79, 0.93)** |
| Knew HbA1c goal |  |  |  |  |
| No | 7.3 (7.2, 7.4) | 0 (ref) | 56.0 (52.6, 59.4) | 1 (ref) |
| Yes | 7.5 (7.3, 7.6) | 0.15 (0.03, 0.28) | 47.4 (43.3, 51.6) | **0.86 (0.80, 0.94)** |
|  | **Systolic blood pressure** | | | |
| Systolic blood pressure, mm Hg | 130.0 (129.0, 131.0) |  | 73.6 (70.7, 76.4) |  |
| Knew recent systolic BP |  |  |  |  |
| No | 129.5 (128.1, 131.0) | 0 (ref) | 75.1 (71.7, 78.3) | 1 (ref) |
| Yes | 130.2 (129.0, 131.4) | -0.69 (-2.22, 0.85) | 73.0 (69.3, 76.5) | 1.00 (0.94, 1.06) |
| Knew systolic BP goal |  |  |  |  |
| No | 129.6 (128.4, 130.8) | 0 (ref) | 74.2 (70.8, 77.4) | 1 (ref) |
| Yes | 130.5 (128.9, 132.0) | 0.56 (-1.02, 2.13) | 72.9 (68.2, 77.2) | 0.97 (0.84, 1.13) |
|  | **Diastolic blood pressure** | | | |
| Diastolic blood pressure, mm Hg | 70.6 (70.0, 71.2) |  | 73.6 (70.7, 76.4) |  |
| Knew recent diastolic BP |  |  |  |  |
| No | 70.3 (69.4, 71.2) | 0 (ref) | 74.7 (71.4, 77.8) | 1 (ref) |
| Yes | 70.8 (70.0, 71.5) | 1.41 (0.26, 2.56) | 73.2 (69.5, 76.6) | 1.01 (0.95, 1.07) |
| Knew diastolic BP goal |  |  |  |  |
| No | 70.4 (69.6, 71.3) | 0 (ref) | 74.1 (70.8, 77.3) | 1 (ref) |
| Yes | 70.9 (70.1, 71.7) | 0.62 (-0.58, 1.82) | 72.9 (68.0, 77.4) | 0.97 (0.83, 1.13) |
|  | **LDL-cholesterol** | | | |
| LDL-cholesterol, mg/dL^†^ | 102.0 (99.0, 104.9) |  | 54.1 (49.6, 58.5) |  |
| Knew recent LDL-cholesterol level |  |  |  |  |
| No | 101.7 (98.1, 105.3) | 0 (ref) | 57.2 (38.7, 74.3) | 1 (ref) |
| Yes | 103.2 (94.1, 112.2) | 4.59 (-4.36, 13.54) | 54.8 (42.9, 66.4) | 1.93 (0.60, 1.44) |
| Knew LDL-cholesterol goal |  |  |  |  |
| No | 101.3 (98.0, 104.6) | 0 (ref) | 55.1 (36.2, 73.0) | 1 (ref) |
| Yes | 105.9 (96.1, 115.7) | 6.84 (-5.90, 15.06) | 48.6 (33.7, 63.8) | 0.73 (0.41, 1.32) |

BP: blood pressure; CI: confidence interval; HbA1c: glycated hemoglobin A1c; LDL: low-density lipoprotein.

The levels and control prevalence are reported for the respective risk factor.

Controlled HbA1c: HbA1c <7%.

Controlled BP: systolic/diastolic blood pressure < 140/90 mm Hg.

Controlled LDL cholesterol: LDL-cholesterol <100 mg/dL.

*Adjusted for age and sex.

^†^Analytic population includes only participants in the fasting subsample, N=950.

Supplemental Table 3. Mean (95% confidence interval) and percentage control for reported knowledge of recent HbA1c level and goal and their associations with mean levels and control of HbA1c among US adults with diabetes stratified by duration of diabetes.

|  | Mean level  (95% CI) | Adjusted difference* (95% CI) | Percentage with control  (95% CI) | Adjusted prevalence ratio for control* (95% CI) |
| --- | --- | --- | --- | --- |
| **Duration of diabetes diagnosis <10 years** |  |  |  |  |
| HbA1c, % | 7.2 (7.1, 7.4) |  | 58.1 (53.9, 62.2) |  |
| Knew recent HbA1c level |  |  |  |  |
| No | 7.2 (7.0, 7.4) | 0 (ref) | 59.6 (54.5, 64.5) | 1 (ref) |
| Yes | 7.2 (7.1, 7.4) | 0.05 (-0.14, 0.25) | 56.7 (51.4, 61.9) | 0.94 (0.84, 1.05) |
| Knew HbA1c goal |  |  |  |  |
| No | 7.2 (7.0, 7.4) | 0 (ref) | 59.9 (55.1, 64.6) | 1 (ref) |
| Yes | 7.3 (7.1, 7.4) | 0.07 (-0.12, 0.27) | 56.0 (50.3, 61.6) | 0.92 (0.81, 1.05) |
| **Duration of diabetes diagnosis ≥10 years** |  |  |  |  |
| HbA1c, % | 7.5 (7.4, 7.6) |  | 44.9 (41.2, 48.5) |  |
| Knew recent HbA1c level |  |  |  |  |
| No | 7.4 (7.3, 7.6) | 0 (ref) | 51.0 (46.4, 55.6) | 1 (ref) |
| Yes | 7.5 (7.4, 7.7) | 0.02 (-0.16, 0.20) | 41.5 (36.4, 46.7) | 0.90 (0.81, 1.00) |
| Knew HbA1c goal |  |  |  |  |
| No | 7.4 (7.2, 7.5) | 0 (ref) | 51.2 (46.3, 56.1) | 1 (ref) |
| Yes | 7.6 (7.5, 7.7) | 0.13 (-0.03, 0.30) | 40.5 (35.3, 45.9) | **0.86 (0.76, 0.98)** |

CI: confidence interval; HbA1c: glycated hemoglobin A1c.

The levels and control prevalence are reported for the respective risk factor.

Controlled HbA1c: HbA1c <7%.

*Adjusted for age and sex.

Supplemental Table 4. Mean (95% confidence interval) and percentage control for reported knowledge of recent BP level and goal, and their associations with mean levels and control of BP among US adults with diabetes stratified by hypertension status. (HTN and BP control: SBP/DBP <140/90 mmHg)

|  | Mean level  (95% CI) | Adjusted difference* (95% CI) | Percentage with control  (95% CI) | Adjusted prevalence ratio for control* (95% CI) |
| --- | --- | --- | --- | --- |
| **Without hypertension** |  |  |  |  |
| Systolic blood pressure, mm Hg | 120.0 (119.1, 121.0) |  |  |  |
| Knew recent systolic BP |  |  |  |  |
| No | 119.6 (118.2, 121.0) | 0 (ref) | 100% | 1 (ref) |
| Yes | 120.4 (119.1, 121.7) | -0.08 (-2.04, 1.89) | 100% | NA |
| Knew systolic BP goal |  |  |  |  |
| No | 119.8 (118.8, 120.9) | 0 (ref) | 100% | 1 (ref) |
| Yes | 120.6 (118.4, 122.8) | 0.42 (-1.92, 2.76) | 100% | NA |
| Diastolic blood pressure, mm Hg | 69.5 (68.5, 70.6) |  |  |  |
| Knew recent diastolic BP |  |  |  |  |
| No | 69.7 (68.1, 71.2) | 0 (ref) | 100% | 1 (ref) |
| Yes | 69.4 (68.1, 70.7) | 1.22 (-0.47, 2.91) | 100% | NA |
| Knew diastolic BP goal |  |  |  |  |
| No | 69.4 (68.2, 70.5) | 0 (ref) | 100% | 1 (ref) |
| Yes | 69.8 (68.0, 71.7) | 0.79 (-1.03, 2.60) | 100% | NA |
| **With hypertension** |  |  |  |  |
| Systolic blood pressure, mm Hg | 134.3 (133.0, 135.6) |  | 62.0 (58.1, 65.8) |  |
| Knew recent systolic BP |  |  |  |  |
| No | 136.9 (135.4, 138.5) | 0 (ref) | 56.5 (51.8, 61.1) | 1 (ref) |
| Yes | 133.5 (131.9, 135.0) | **-3.44 ( -5.33, -1.55)** | 63.8 (59.1, 68.3) | **1.08 (1.01, 1.15)** |
| Knew systolic BP goal |  |  |  |  |
| No | 135.4 (133.9, 136.8) | 0 (ref) | 58.9 (54.3, 63.3) | 1 (ref) |
| Yes | 133.1 (131.2, 135.0) | -1.86 (-3.88, 0.15) | 65.6 (59.9, 71.0) | 1.16 (1.00, 1.34) |
| Diastolic blood pressure, mm Hg | 70.2 (69.3, 71.1) |  | 62.0 (58.1, 65.8) |  |
| Knew recent diastolic BP |  |  |  |  |
| No | 70.8 (69.6, 72.0) | 0 (ref) | 56.7 (52.2, 61.0) | 1 (ref) |
| Yes | 71.2 (70.3, 72.2) | 0.20 (-1.19, 1.60) | 63.9 (59.2, 68.5) | **1.09 (1.02, 1.16)** |
| Knew diastolic BP goal |  |  |  |  |
| No | 71.1 (69.9, 72.2) | 0 (ref) | 59.1 (54.6, 63.4) | 1 (ref) |
| Yes | 71.2 (70.1, 72.2) | -0.56 (-2.19, 1.07) | 65.6 (59.7, 71.1) | 1.15 (0.99, 1.34) |

*Adjusted for age and sex.

BP: blood pressure; CI: confidence interval.

The levels and control prevalence are reported for the respective risk factors.

Controlled BP: systolic and diastolic blood pressure < 140 and <90 mm Hg.

NA – not applicable. Prevalence ratios for blood pressure control were not estimated for those without hypertension since all participants without hypertension had systolic and diastolic blood pressure < 140 and <90 mm Hg.

Supplemental Table 5. Estimation of mean and percentage control for being aware of recent LDL-cholesterol value and goal and their associations with mean levels and control of LDL-cholesterol among US adults with diabetes stratified by statin use.

|  | Mean level  (95% CI) | Adjusted difference* (95% CI) | Percentage with control  (95% CI) | Adjusted prevalence ratio for control* (95% CI) |
| --- | --- | --- | --- | --- |
| **Not taking statin** |  |  |  |  |
| LDL-cholesterol, mg/dL^†^ | 119.8 (115.1, 124.5) |  | 31.8 (25.0, 39.2) |  |
| Knew recent LDL-cholesterol level |  |  |  |  |
| No | 118.7 (113.4, 124.0) | 0 (ref) | 33.4 (26.0, 41.4) | 1 (ref) |
| Yes | 130.0 (118.2, 141.9) | 12.13 ( -1.03, 25.30) | 17.3 (5.9, 35.8) | 0.46 (0.16, 1.28) |
| Knew LDL-cholesterol goal |  |  |  |  |
| No | 119.6 (114.2, 125.0) | 0 (ref) | 32.5 (25.5, 40.2) | 1 (ref) |
| Yes | 122.4 (109.1, 135.7) | 1.75 (-13.87, 17.36) | 23.8 (8.0, 47.7) | 0.75 (0.24, 2.38) |
| **Taking statin** |  |  |  |  |
| LDL-cholesterol, mg/dL^†^ | 89.7 (86.0, 93.4) |  | 69.5 (63.3, 75.3) |  |
| Knew recent LDL-cholesterol level |  |  |  |  |
| No | 88.2 (84.8, 91.6) | 0 (ref) | 70.4 (64.6, 75.8) | 1 (ref) |
| Yes | 94.8 (86.0, 103.6) | 7.15 (-0.05, 14.36) | 66.5 (52.2, 78.8) | 0.85 (0.55, 1.33) |
| Knew LDL-cholesterol goal |  |  |  |  |
| No | 87.2 (83.8, 90.7) | 0 (ref) | 72.4 (66.2, 77.9) | 1 (ref) |
| Yes | 101.8 (90.9, 112.8) | **14.39 (5.34, 23.43)** | 55.7 (36.7, 73.6) | 0.54 (0.28, 1.05) |

^†^Analytic population includes only participants in the fasting subsample, N=950.

LDL: low-density lipoprotein.

The levels and control prevalence are reported for the respective risk factors.

Controlled LDL cholesterol: LDL-cholesterol <100 mg/dL.

*Adjusted for age and sex.

^†^Analytic population includes only participants in the fasting subsample, N=950.
